# Optimization of Photosynthetic Productivity in Contrasting Environments by Regulons Controlling Plant Form and Function

**DOI:** 10.3390/ijms19030872

**Published:** 2018-03-15

**Authors:** Barbara Demmig-Adams, Jared J. Stewart, Christopher R. Baker, William W. Adams

**Affiliations:** 1 Department of Ecology & Evolutionary Biology, University of Colorado, Boulder, CO 80309-0334, USA; jared.stewart@colorado.edu (J.J.S.), william.adams@colorado.edu (W.W.A.); 2 Department of Plant & Microbial Biology, University of California, Berkeley, CA 94720-3102, USA; cbaker@berkeley.edu

**Keywords:** acclimation, adaptation, *Arabidopsis thaliana*, C-repeat binding factor (CBF), dehydration-responsive element-binding (DREB), photosynthesis, source–sink, transcription factors

## Abstract

We review the role of a family of transcription factors and their regulons in maintaining high photosynthetic performance across a range of challenging environments with a focus on extreme temperatures and water availability. Specifically, these transcription factors include CBFs (C-repeat binding factors) and DREBs (dehydration-responsive element-binding), with CBF/DREB1 primarily orchestrating cold adaptation and other DREBs serving in heat, drought, and salinity adaptation. The central role of these modulators in plant performance under challenging environments is based on (i) interweaving of these regulators with other key signaling networks (plant hormones and redox signals) as well as (ii) their function in integrating responses across the whole plant, from light-harvesting and sugar-production in the leaf to foliar sugar export and water import and on to the plant’s sugar-consuming sinks (growth, storage, and reproduction). The example of *Arabidopsis* *thaliana* ecotypes from geographic origins with contrasting climates is used to describe the links between natural genetic variation in CBF transcription factors and the differential acclimation of plant anatomical and functional features needed to support superior photosynthetic performance in contrasting environments. Emphasis is placed on considering different temperature environments (hot versus cold) and light environments (limiting versus high light), on trade-offs between adaptations to contrasting environments, and on plant lines minimizing such trade-offs.

## 1. Introduction

Photosynthesis converts sunlight into food, plant-derived materials, and carbon-based fuels. Continuing human population growth increases the demand for these necessities, while extreme climate events threaten crop productivity. It is thus pivotal to maintain or increase plant productivity, and specific targets to achieve this goal include improvement of rates and efficiency of photosynthesis [1,2]. Plants fine-tune their investment in photosynthetic capacity in response to environmental conditions; this review focuses on temperature, water availability, and sunlight. Improving crop yields as climate changes will require a deepened mechanistic understanding of plant features supporting high plant productivity under challenging environmental conditions. Many studies on temperature effects have focused on the extremes of lethal freezing or heat (e.g., [3,4]) and on damage from membrane disruption and leakage (e.g., [5,6]). However, the non-lethal temperatures between these extremes profoundly impact photosynthetic performance and plant productivity. Here, we review plant features that support high photosynthetic productivity over a range of temperature and light environments. We end with a synopsis of recently identified trade-offs in the ability of locally adapted populations of the winter annual model species *Arabidopsis thaliana* to maintain photosynthetic productivity in contrasting environments. This concluding section focuses on selected transcription factors, signaling networks, and the foliar vasculature’s role in exchanging sugar and water between the photosynthesizing leaf and the rest of the plant. We hope that this insight will be helpful in efforts to improve crops for enhanced productivity under a changing and increasingly unpredictable climate.

**Figure 1:**
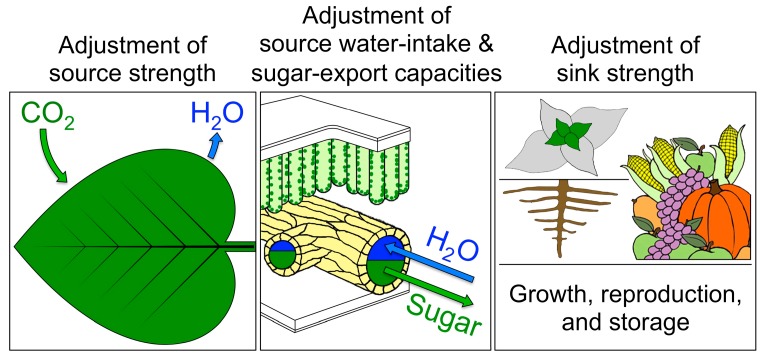
Schematic depiction of the three proposed adjustments necessary for plant productivity in a given environment: (i) photosynthetic capacity (light- and CO_2_-saturated intrinsic capacity of photosynthesis determined by the level of photosynthetic proteins) to provide sugars (the plant’s source strength) fueling crop yield; (ii) high flux capacities for distributing water throughout the leaf as well as loading and exporting sugars from the leaf (foliar water-intake & sugar-export capacities; see Section 4.5 below); and (iii) sink activity (growth, reproduction, and storage; sink strength). Modified from Demmig-Adams et al. [7] and Adams et al. [10]. The left panel illustrates CO_2_ uptake and water loss through leaf stomates, which requires sufficient water delivery to the leaf. The middle panel shows a leaf vein that contains conduits for water import (blue) into and conduits for sugar export (green) out of the leaf. The right panel shows examples of sugar-consuming and -storing sink tissues that constitute the plant’s demand for photosynthate.

High plant productivity in any given environment depends on high activities of three key functions (Figure 1): (i) photosynthetic capacity of source leaves; (ii) the capacities of the leaf’s sugar-exporting and water-importing vascular pipelines; and (iii) activity of plant sinks (sum of growth, reproduction, and storage). Source leaf photosynthesis is the plant’s engine (see [7]). Sugar demand from plant sinks influences the expression of photosynthetic genes [8]; sufficient foliar sugar-export capacity is needed to prevent protracted foliar sugar accumulation and photosynthetic gene repression [9,10]. Moreover, adequate foliar water-import capacity must replace water lost during carbon uptake to ensure continued stomatal opening and access to atmospheric carbon dioxide [11,12].

The intrinsic photosynthetic capacity is adjusted in response to the combined sugar demand from all sink tissues, with a high photosynthetic capacity when total sink activity is high [7,8]. Vegetative growth of shoots and/or roots is one possible sink and trigger for photosynthetic upregulation, while storage of photosynthate and reproduction are alternative key sinks that can also support high photosynthetic capacity. Therefore, plant productivity is the sum-total of vegetative growth, energy storage, and flower and seed/fruit production. In fact, strong increases in the yield of grain/fruit crops were achieved in numerous instances by the generation of dwarf varieties or pruning practices that led to an increased allocation of photosynthate to grain/fruit yield relative to vegetative growth (reviewed, e.g., in [7]).

The above functions (photosynthetic capacity, sugar- and water-transport capacity, and sink strength) are adjusted in response to the environment by orchestrated changes at the transcriptomic, metabolic, and phenotypic levels. The present review provides an overview of changes at these different levels, including selected transcription factors that co-optimize plant productivity and tolerance to cold, heat, and drought (Section 2), selected metabolic change (example of redox regulation; Section 3), and a case study of associated changes in plant form and function that support superior photosynthetic productivity under extreme temperatures (Section 4). Section 4 also illustrates genetic differences between locally adapted plant populations in the degree of these adjustments.

This insight underscores the need for a whole-plant perspective and integration of genomic and phenotypic information to develop plant genotypes with superior productivity. Progress in “profiling crop germplasm [that] has benefited from rapid advances in DNA sequencing” now needs to be matched with “similar advances [in] the throughput of plant phenotyping” [13]. We suggest that proxies for the leaf features reviewed here, including those responsible for foliar water import and sugar export, merit attention in the development of corresponding markers suitable for high-throughput phenotyping.

## 2. Transcription Factors Co-Optimize Productivity and Stress Tolerance

### 2.1. CBFs/DREBs and Abiotic Stress Tolerance

The present review focuses on a set of selected transcription factors with roles in maintaining high photosynthetic performance in extreme environments, i.e., the C-repeat binding factor (CBF)/dehydration-responsive element-binding (DREB) family (for multiple prior reviews on this topic, see below). The centrality of these gene regulators for maintaining plant performance under challenging environments is based on their integrative coordination of multiple aspects of plant form and function as well as their interaction with central signal-transduction networks that link environmental cues to the transcriptome. These transcription factors coordinate multiple morphological, physiological, and biochemical adjustments across the plant (e.g., [14])—from light collection and sugar production in source leaves to the activity of sugar-consuming sinks (growth, storage, and reproduction) [15,16,17]. In addition, these transcription factors show cross-talk with key signaling networks that sense environmental cues [18], which makes these transcription factors subject to modulation by the environment.

CBFs/DREBs regulate the expression of genes responding to both low and high temperatures in *A. thaliana* [3,19] and other species (e.g., [20]; for reviews, see [18,21,22,23,24]). One branch of this transcription factor family serves primarily in orchestrating cold adaptation and the other primarily in orchestrating heat, drought, and salinity adaptation. Specifically, CBF1, 2, and 3—also known as DREB1B, C, and A, respectively—are prominent regulators of plant growth and development under cold temperature [25], while DREB2 transcription factors regulate the response to heat and drought [19,26], and CBF4/DREB1D and DREB3 are implicated in ABA-dependent drought response (e.g., [27,28]). Despite these specialized roles, CBF/DREB1-type and DREB2-type transcription factors have additional overlapping functions in temperature adaptation [24].

The CBF transcription factors, furthermore, act on other transcriptional modulators, such as the regulator *ICARUS1* of growth under high temperature in *A. thaliana* [29], and interact with plant hormones in coordinating growth under temperature extremes [30]. Moreover, Kurbidaeva et al. [31] demonstrated a role for natural genetic diversity in the *INDUCER OF CBF EXPRESSION 2* gene in freezing tolerance among sixty *A. thaliana* ecotypes. While the evidence linking CBFs and freezing tolerance in *A. thaliana* is substantial [32,33,34,35,36], CBFs’ role in orchestrating above-freezing, cool-temperature response may be even more important [37,38,39,40,41]. Specifically, the CBF regulon has been implicated in plant cool-temperature response involving thicker leaves with more chlorophyll and higher rates of photosynthesis per leaf area in *A. thaliana*, and constitutive overexpression of CBF transcription factors yielded the same phenotype in plants grown under moderate temperatures [14,42,43].

### 2.2. Stress-Inducible Promoters: On-off Switches Minimize Yield Penalties

A comprehensive review by Agrawal et al. [24] on the CBF/DREB system lists studies on transgenic expression of *CBF* or other *DREB* genes in crops that produced gains in stress tolerance at the expense of growth retardation and yield penalties under optimal environmental conditions. Next, the latter review examined additional studies that used different promoters and achieved enhanced stress tolerance free of penalties under optimal conditions [26,28,44,45,46]. Agarwal et al. [24] concluded that “it is possible to decrease or eliminate negative influences of CBF/DREB-encoding transgenes on plant growth and yield by modulation of their levels of expression, through the use of stress-inducible promoters.” In other words, stress-inducible promoters assist in combining high plant productivity under favorable conditions with high stress tolerance.

**Figure 2:**
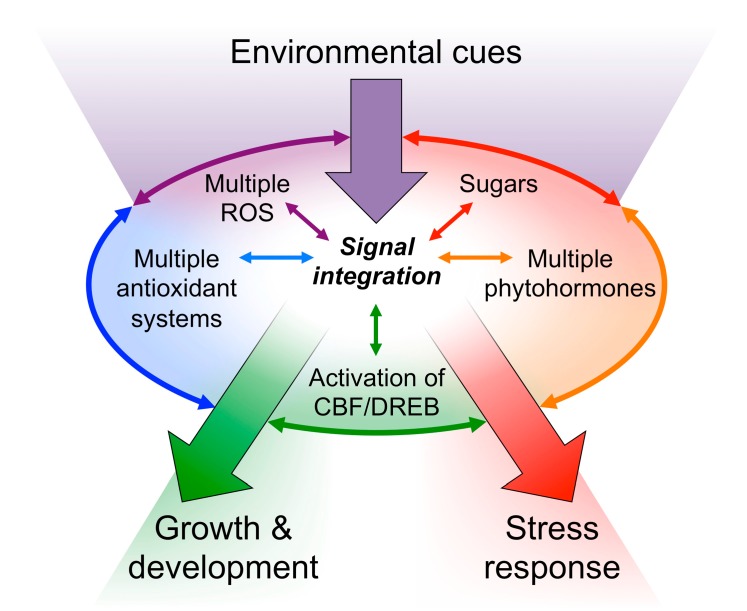
Schematic depiction of prominent signaling pathways involved in translating environmental cues (e.g., light and temperature) into phenotypic responses.

Switching the CBF/DREB regulons on or off via stress-inducible promoters involves cross-talk among plant hormones, cellular redox balance (the balance between stress-induced generation of reactive oxygen species [ROS], and the plant’s production of antioxidants), and other messengers ([17,24]; see Figure 2). Reactive oxygen species, antioxidants, phytohormones, and sugars interact in the transduction of environmental cues into modulation of stress tolerance, programmed cell death, growth, and development [47,48]. Trends are complex due to local gradients in ROS and antioxidants, multiple types of ROS and antioxidant systems with different, sometimes antagonistic, effects, and the impacts of both environmental stimuli and genetic factors [47,48,49]. Redox signals modulate plant hormone synthesis, sequestration, and signal transduction. Phytohormones subject to redox modulation include abscisic acid, auxins, brassinosteroids, cytokinins, gibberellins, ethylene, salicylic acid, and jasmonic acid [16,47,48,49,50,51].

Reactive oxygen species have a dual role in cell expansion and growth. Under environmental stress, ROS can either stimulate or inhibit leaf and root cell expansion, depending on the specific ROS involved, their location, and other conditions [52,53]. More studies are needed to further elucidate interactions among transcription factors, oxidants/antioxidants, phytohormones, and sugar signals in the modulation of plant growth, photosynthetic performance under challenging environmental conditions, and the foliar vasculature responsible for water and sugar transport.

### 2.3. To Grow or Not to Grow When the Going Gets Tough

As mentioned in Section 2.2, stress-inducible promoters can minimize growth penalties under optimal conditions. In addition, growth cessation is not the only possible response to stressful conditions. In fact, either slowed growth or enhanced growth can be adaptive when plants face water shortages and/or extreme temperatures, depending on the specific environmental conditions and plant genetic background. In reference to this conundrum, a review by Dolferus [54] on abiotic stress tolerance is entitled, “To grow or not to grow: a stressful decision for plants.” However, despite these alternative responses depending on specific circumstances, some general trends in plant growth under either cold temperature or heat/drought can be identified. One ubiquitous plant response in a diversity of stressful environments is increased ROS production [55]. As stated above, ROS can either stimulate or inhibit growth, and plant growth itself can likewise either be accelerated or stunted in stressful environments. For example, in montane ecosystems with frozen soils, evergreens typically arrest growth and downregulate photosynthesis during winter [56,57]. This downregulation of photosynthesis is helpful to prevent the opening of stomates on intermittent winter days with mild air temperatures when there is no opportunity to replace water lost from leaves or needles. In contrast to these evergreens that persist throughout entire harsh seasons in a state of suspended activity, herbaceous biennials and winter annuals grow only where soil water is available on intermittently warm days, upregulate photosynthetic capacity under cool temperature, and take full advantage of mild winter days to produce carbohydrates for immediate or future growth. Similar contrasts in growth responses are seen in desert environments (see [58]). Many evergreens arrest growth and photosynthesis as summer heat/drought set in. In contrast, desert ephemerals, which germinate and grow rapidly during a short rainy season, accelerate growth and complete their lifecycle before drought sets in.

### 2.4. CBFs and the Phenotype of Cool-Grown Winter Annuals

Under cool conditions in autumn and winter, winter annuals typically slow their vegetative growth and upregulate photosynthetic capacity [7,15,16,17]. This upregulation of photosynthetic capacity allows maintenance of photosynthesis under prevailing cool temperature, and record rates of photosynthesis under warm temperature (see [59]). This photosynthetic upregulation in overwintering herbaceous species allows continued carbon gain in winter, supporting exceptionally high photosynthesis rates during intermittent warm, high-light periods, and thereby contributing to accelerated growth and flowering in the spring.

Hüner et al. [16] discussed the cool-temperature-induced photosynthetic upregulation of winter annuals in the context of the CBF regulon, and suggested that enhancing the capability for bursts of photosynthetic activity in crops would accelerate growth during the spring season with moderately cool temperatures, high water availability, and high water-use efficiency. Early sowing of summer annuals has been shown to provide relief by improving water availability [60] as well as seed yield and quality [61] in geographic areas with intense summer heat and drought. However, moving agriculture to higher latitude or altitude with less summer heat and drought, or early sowing of summer crops, exposes these plants to periods of low temperature. The benefits of early sowing of summer annuals might thus be enhanced by targeting the CBF/DREB system in efforts to improve photosynthetic performance under cool temperature.

### 2.5. Example of a Gene Capable of Orchestrating either Reduced or Accelerated Growth under Heat/Drought

As stated above, plant adaptation to hot/dry conditions can involve either reduced growth or accelerated growth and early flowering. One gene involved in both of these strategies, the *FRIGIDA* (*FRI*) gene, was studied by Lovell et al. [62] in *A. thaliana*. Low *FRI* expression was associated with a strategy of drought escape, consisting of rapid growth and early flowering, allowing for completion of the life cycle before peak drought/heat. On the other hand, high *FRI* expression was associated with slowed growth, reduced use of water per plant, and delayed flowering. The control of flowering time also involves a number of other genes (see [63]), the discussion of which is beyond the scope of this review.

## 3. Redox Regulation and Photoprotection

### 3.1. The Chloroplast Transduces the State of the Environment into Redox Signals

The redox-signaling networks that modulate plant growth and development receive input from plant sensors that gauge opportunity or threat posed by the environment [47,48,49]. In the green leaf, the chloroplast plays a key role in sensing limitations posed by unfavorable temperatures or light intensities, drought, and other conditions as described below. Any and all of these environmental conditions affect the chloroplast’s balance between absorbed and utilized light energy. Limitations to photosynthesis or plant growth lower the fraction of absorbed light that can be utilized in these processes, and thereby increase the level of excess absorbed light. The resulting excess excitation energy can produce ROS, which provides input into the cellular redox-signaling network [64,65]. Among the targets of redox modulation are the CBF/DREB regulons (see Section 2.2).

The chloroplast’s many antioxidation systems modulate the level of excess light, ROS production, and redox signal generation. For example, photoprotective, preemptive thermal dissipation of excess excitation energy and ROS-detoxifying antioxidants like tocopherols lower the levels of ROS and their derivatives [66]. In Section 4, we describe ecotypic differences in the capacity for thermal energy dissipation and foliar tocopherol levels between locally adapted *A. thaliana* ecotypes grown under common conditions, which indicates genetic differences in the extent of redox-signal generation.

### 3.2. Redox Signals Modulate Vascular Infrastructure

Studies with tocopherol-deficient mutants have established a link between tocopherol-dependent signaling and the foliar vasculature [67,68,69,70,71,72]. Tocopherol levels affected the specific organization of water and sugar conduits (Table 1), but impacted neither total sugar- and water-transport capacity nor photosynthetic capacity (not shown; see [72]). Tocopherol-deficient mutant had more but narrower water conduits than the Col-0 wild-type (Table 1; [72]), which would be expected to decrease cavitation risk under high evaporative demand, thereby increasing high-temperature tolerance [73,74] and maintaining stomatal opening [75,76]. These adjustments thus bolstered high-temperature tolerance with no penalty for photosynthesis. It is possible that plant lines with augmented tocopherol levels would be more prone to cavitation events under high temperature. Efforts to engineer plant antioxidant levels need to take such responses into consideration.

It should be noted that vascular organization may not only affect plant tolerance to abiotic stress, but also to the many pathogens that spread through the vasculature (such as viruses, bacteria, and fungi; [77,78,79]). Modulation of foliar vascular organization thus has the potential to serve in co-optimizing plant productivity and abiotic and biotic stress tolerance.

### 3.3. Manipulation of Thermal Energy Dissipation and Plant Growth: Photon-Capture Efficiency and Redox Regulation

Leaves exposed to full sunlight absorb more light than they can utilize in photosynthesis, and the potentially harmful excess excitation energy is dissipated via thermal energy dissipation, triggered by increased trans-thylakoid pH (ΔpH; [80,81,82]). When the light level absorbed by the leaf is no longer excessive and no other stresses are present, thermal dissipation is disengaged via a drop in ΔpH and a removal of the xanthophyll pigments involved in dissipation. Leaves engineered for accelerated ΔpH abolishment [83] or accelerated removal of energy-dissipating xanthophylls [84] exhibited increased carbon uptake after transitions from high to low light and accumulated significantly more biomass.

Future research should elucidate to what extent this enhanced biomass production is due to increased energy availability as a result of increased photon-capture efficiency, and to what extent growth stimulation may result from increased production of ROS signals that stimulate cell expansion and growth (see above). An association between accelerated plant growth and a decreased emphasis on thermal dissipation and increased emphasis on ROS production is consistent with findings by Esteban et al. [85]. The latter authors conducted a large survey among photosynthetic organisms and revealed an evolutionary trend towards decreased emphasis on thermal energy dissipation by xanthophylls. This trend represents a progression away from preemptive removal of excitation energy towards permitting greater ROS production. Such a progression would speed up return to maximal photon-capture efficiency upon transition to limiting light as well as allow greater levels of growth-stimulating ROS. Comparative eco-physiological studies have identified a greater emphasis on pre-emptive thermal energy dissipation in perennials (and particularly evergreens) compared to annual species [66]. For slow-growing perennials, the penalty of a somewhat lower photon-capture efficiency upon return to limiting light may be minimal, whereas faster-growing annuals with higher growth rates may derive particularly pronounced benefit from an accelerated return to maximal photon-capture efficiency [66].

There may also be natural ecotypic variation in thermal energy dissipation kinetics. Two ecotypes of *A. thaliana* from Sweden and Italy vary in the degree to which they adjust to low versus high growth light intensity ([86]; see also Section 4.6 below). The Italian ecotype had a superior ability to acclimate to low growth light intensity and high temperature, exhibiting higher growth rates under both conditions [76,86,87]. Our preliminary observations also indicate that speed and extent of the relaxation of thermal energy dissipation subsequent to high-light exposure are greater in the Italian ecotype [88]. In addition, two genes that speed up transition from high thermal energy dissipation rates to maximal photochemical efficiency were expressed at higher levels in the Italian ecotype under low light and warm temperature [89]. These genes encode zeaxanthin epoxidase, which functions in the removal of dissipating xanthophylls, and the K^+^/H^+^ antiporter KEA3 that helps abolish ΔpH, the overexpression of either of which resulted in greater biomass production in tobacco [83,84].

## 4. Natural Populations Illustrate How Trade-Offs in Performance under Cool versus Hot Temperature Can Be Minimized

### 4.1. Arabidopsis thaliana Ecotypes from Sweden and Italy Show Differential Adaptation Patterns and CBF Expression

The model species *A. thaliana* is a winter annual with a natural geographic range covering climates with very cold to warm temperatures. A comparison of ecotypes from Sweden and Italy illustrates the links between natural genetic variation in CBF transcription factors and differential acclimation of plant features supporting superior photosynthetic performance in contrasting environments. While these two ecotypes originate from different latitudes and temperature regimes (see [75]), their sites of origin are very similar with respect to distance from coast and altitude [90]. These ecotypes differ in their acclimatory responsiveness to growth temperature environment (hot versus cold) and light environment (limiting versus high light), and especially in the extent to which they switch off the trademark cool-temperature/high-light phenotype of winter annuals when grown under common controlled conditions, as is detailed below. Moreover, in their respective native habitats, the Italian ecotype grows rapidly and flowers early in spring, whereas the Swedish ecotype grows and flowers later in early summer [90] (for a host of other phenotypic differences, see below). This difference in flowering time between the ecotypes can be viewed in the context of the accelerated growth and flowering discussed in Section 2.3 for genotypes adapted to habitats with warm/dry summers.

CBF2/DREB1C was initially thought to negatively regulate *CBF1*/*DREB1B* and *CBF3*/*DREB1A* expression and reduce freezing tolerance in *A. thaliana* [91]. However, recent evidence suggests CBF2 may, in fact, be more important for cold acclimation than CBF1 and CBF3 (see, e.g., [92] and Discussion therein). These latter findings are consistent with those from studies on wild populations of *A. thaliana*. The CBF2 transcription factor is nonfunctional in the Italian ecotype [93], as well as in a number of other *A. thaliana* ecotypes from warm climates ([32,34]; see also [94]). Reduced tolerance of the Italian ecotype to deep-freeze events compared to the Swedish ecotype has been linked to the Italian ecotype’s nonfunctional CBF2 through both quantitative trait loci mapping ([35]; see also [95]) and transgenic [93] approaches. However, the Italian ecotype does exhibit substantial upregulation of photosynthetic capacity under cool temperature [75,76] and, furthermore, switches off the cool-temperature/high-light phenotype more effectively than the Swedish ecotype under either hot temperature or low light ([76,86,87,96]; for details, see below). Over a considerable range of growth temperatures between 8 °C and 35 °C, the Italian ecotype is thus able to minimize trade-offs.

### 4.2. The Cool-Temperature Phenotype of Winter Annuals Is Reminiscent of a High-Light Phenotype

Some of the mechanisms that underlie acclimation to cold temperature in winter annuals also underlie acclimation to high light [15,17,97]. In plants grown under either cool temperature or high light, the Swedish ecotype exhibits thicker leaves, higher chlorophyll levels, and a higher photosynthetic capacity on a leaf area basis compared to the Italian ecotype.

Concomitant increases of the number of chloroplast-rich palisade layers and leaf vascular capacity led to the coordinated upregulation of light collection, carbon uptake, and sugar export under cool growth temperature and/or high growth light intensity [71,75,98]. The exceptionally high photosynthetic capacity in cool-grown plants of the Swedish ecotype [59,75,76] is conducive for taking advantage of narrow windows of opportunity for carbon gain during warm, high-light periods during mild winter and spring days.

### 4.3. An Experimental Design Suitable to Identify On-Off Switches That Minimize Penalties

**Figure 3:**
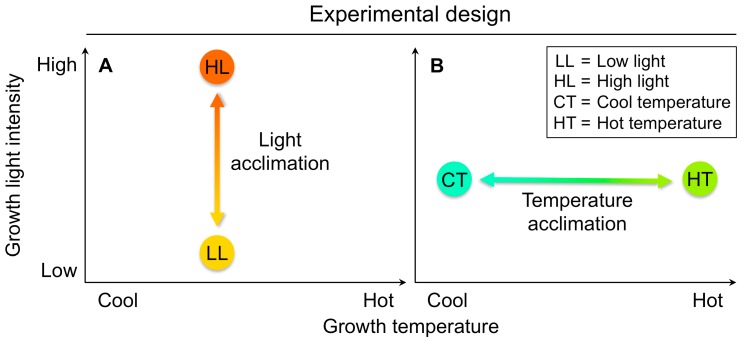
Schematic depiction of the experimental design used to assess (**A**) light acclimation and (**B**) temperature acclimation of phenotypic traits (see e.g., Stewart et al. [71]). LL, HL—growth light intensities of 100 and 1000 µmol photons m^−2^·s^−1^, respectively, at a leaf temperature of 20 °C during the light period; CT, HT—leaf temperatures of 14 °C and 36 °C, respectively, under a growth light intensity of 400 µmol photons m^−2^·s^−1^.

To be able to identify on/off switches and their genetic basis, experimental design must test for genotypic differences in the degree of responsiveness to environmental conditions. In order to quantify the effect of genetic adaptation on phenotypic plasticity, genotypes must be compared under more than a single environmental condition. Choosing contrasting points on the spectrum of nonlethal conditions is helpful to reveal ecotypic differences in the ability to switch responses on and off. To compare the extent of their adjustment to different temperature and light environments, the Italian and Swedish ecotypes were grown under low versus high light (under moderate growth temperature), and also under cool versus hot temperature (under moderate growth light intensity; Figure 3). As detailed below, this approach revealed a host of informative ecotypic differences in the degree of up- or downregulation of key plant features and associated genes.

An additional aspect of experimental design concerns plant source-sink balance. Ecotypic differences in phenotypic adjustment in anatomy, morphology, and photosynthetic performance would be expected to be masked by plant growth under sink-limiting conditions that do not permit full expression of potential plant activity under the environmental conditions of interest. Growth of plants in large rooting volumes with an adequate supply of water and nutrients, and resulting low levels of sink limitation, facilitates identification of ecotypic differences in photosynthetic capacity (see discussion in [75,76]).

The following sections present a synopsis of findings from our group’s approach to identify trade-offs between adaptation to low versus high growth light intensity and cold versus hot growth temperature. We review how these trade-offs can be minimized in the example of the Swedish and Italian ecotypes of *A. thaliana*, with attention to plant growth, involvement of redox state and CBF, and the foliar vasculature’s role in facilitating whole-plant source-sink relationships and optimizing photosynthetic performance.

### 4.4. Differential Adjustment of Leaf Form and Function in Swedish and Italian Ecotypes

While the Italian ecotype adjusts effectively to hot-temperature or low-light environments by switching off the trademark leaf-morphological responses governing acclimation to cool temperature or high light in this winter annual, the Swedish ecotype exhibits reduced growth under both conditions [76,86]. The Italian ecotype thereby minimizes growth penalties in low-light or hot-temperature environments.

#### 4.4.1. Different Growth Rates in Swedish and Italian Ecotypes

**Figure 4:**
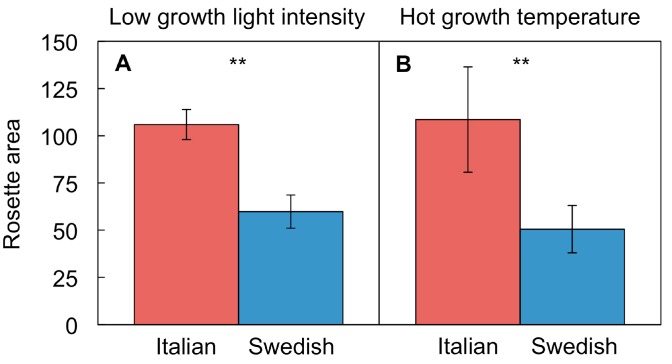
Rosette area (total light-exposed leaf area per plant) of Italian and Swedish ecotypes of *A. thaliana* grown (**A**) under low light intensity (100 µmol photons m^−2^·s^−1^) at a moderate leaf temperature (20 °C) and (**B**) at a hot leaf temperature (36 °C) under moderate light intensity (400 µmol photons m^−2^·s^−1^). Mean values ± standard deviations (*n* = 3 to 4); significant differences (*t*-test) indicated by asterisks—** = *p <* 0.01. Data from Stewart et al. (**A**) [86] and (**B**) [76]. The latter references also include images of plants grown under the various conditions.

Much smaller rosettes (Figure 4) with lower biomass are produced by the Swedish versus the Italian ecotype under either hot growth temperature [76] or low growth light intensity [86]. The faster growth of the Italian ecotype under hot temperature could, once again, be viewed as serving in heat/drought escape (see Section 2.3 above).

#### 4.4.2. Differential Antioxidant Levels between Swedish and Italian Ecotypes

Since ROS can stimulate leaf expansion [52], it is of interest whether the Italian ecotype exhibits lower antioxidant levels than the Swedish ecotype. Indeed, the levels of the antioxidant tocopherol are lower in the Italian ecotype under both hot growth temperature and low growth light intensity compared to the Swedish ecotype [76,87]. In addition, the maximal capacity for removal of excess excitation energy via thermal dissipation, which counteracts ROS production, is lower in the Italian ecotype grown under short daily periods of high light [87]. Similarly, Oakley et al. [99] also found lower levels of thermal energy dissipation in the Italian versus the Swedish ecotype upon transfer to cold temperature. Future research should quantify the levels of ROS in the two ecotypes under various growth conditions, and also elucidate the genetic basis of altered antioxidant levels in the Italian ecotype and their link to superior growth and rosette expansion under certain conditions.

#### 4.4.3. Differential *CBF* Expression between Swedish and Italian Ecotypes

**Figure 5:**
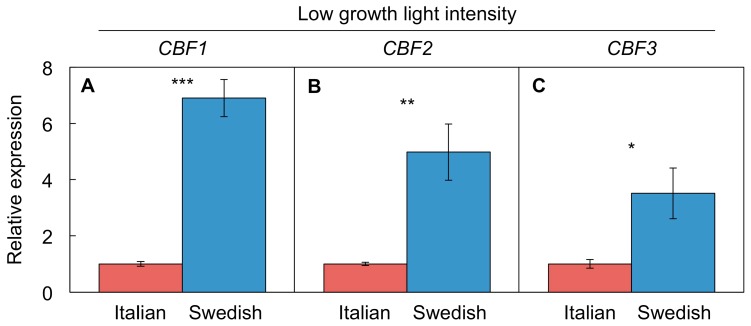
Relative expression (with mean transcript values for the Italian ecotype set to 1) of (**A**) *CBF1* (*AT4G25490*), (**B**) *CBF2* (*AT4G25480*), and (**C**) *CBF3* (*AT4G25470*) genes from leaves of Italian (Castelnuovo-12 [ABRC stock number: CS98761], sub-line 24) and Swedish (Rodasen-47 [ABRC stock number: CS98762], sub-line 29) ecotypes of *A. thaliana* grown under low light intensity (9-h photoperiod of 100 µmol photons m^−2^·s^−1^) at warm temperature (25 °C/20 °C [light/dark] air temperature). Mean values ± standard deviation (*n* = 4); significant differences (*t*-test) indicated by asterisks—* = *p <* 0.05, ** = *p <* 0.01, *** = *p <* 0.001. Leaf discs of 0.73 cm^2^ were homogenized in liquid nitrogen by bead beating and RNA was extracted and DNase-treated (Qiagen RNeasy Plant Mini Kit). cDNA synthesis was performed with 2 μg of DNase-treated RNA per sample (Qiagen Omniscript cDNA Synthesis Kit). Due to the sequence similarity of the three *CBF* genes, qPCR primers were designed using the NCBI Primer-BLAST Tool in order to minimize off-target amplification of paralogous genes. qPCR was performed with 40 ng of cDNA per sample (Applied Biosystem’s Fast Sybr Green Master Mix) and the housekeeping gene *UBC21* (*AT5G25760*) was used as a control.

The known dysfunction of the CBF2 transcription factor in the Italian ecotype is associated with differences in gene expression of *CBF1*, *CBF2*, and *CBF3* (Figure 5). Figure 5 shows relative *CBF1–3* gene expression levels for the Italian versus Swedish ecotype under low growth light intensity and warm temperature, where the rosette-expansion rate is much higher in the Italian ecotype (Figure 4). The higher *CBF1–3* expression under low light in the Swedish ecotype is likely the reason for the stunted growth of this ecotype under this condition. It can be concluded that the Italian ecotype possesses a superior ability to turn off *CBF* gene expression under low light at warm temperature. It should be noted that the *CBF2* gene is still expressed (and responsive to, e.g., cold temperatures) in the Italian ecotype, but its CBF2 protein is not functional due to a mutation that results in a premature stop codon [93].

#### 4.4.4. Ability to Switch Off Other Features of the Cool-Temperature/High-Light Phenotype

**Figure 6:**
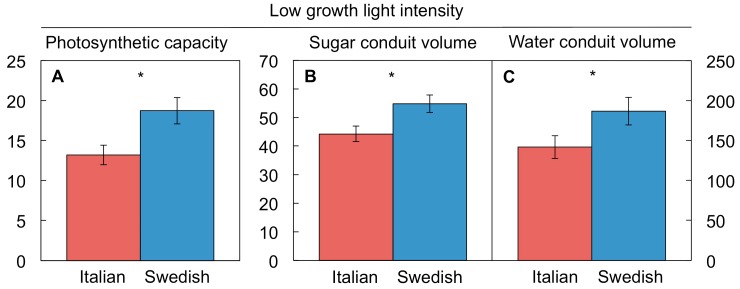
(**A**) Photosynthetic capacity (µmol O_2_ m^−2^·s^−1^) and volumes (mm^3^ m^−2^) of minor-vein (**B**) sugar-transporting and (**C**) water-transporting conduits in leaves of Italian and Swedish ecotypes of *A. thaliana* grown under low light intensity (100 µmol photons m^−2^·s^−1^) at moderate leaf temperature (20 °C). Mean values ± standard deviations (*n* = 4); significant differences (*t*-test) indicated by asterisks—* = *p <* 0.05. Data from Stewart et al. [86].

In addition to the growth features shown above, the Swedish ecotype also constitutively maintains other high-light characteristics in low light, such as a higher photosynthetic capacity and greater volumes of sugar-export and water-import conduits [86,87], while the Italian ecotype is able to switch off this phenotype more effectively (Figure 6). It is attractive to assume that this differential phenotypic adjustment is associated with the differential *CBF* expression and differential antioxidant levels shown above.

### 4.5. A Key Role for Vascular Adjustment: High Photosynthesis Rates Supported by Improved Sugar Removal at Cool Temperature and Improved Water Delivery at Hot Temperature

#### 4.5.1. Coordination of Photosynthetic Productivity with Sugar and Water Transport

A leaf’s photosynthetic capacity is coordinated with the capacity for sugar and water transport ([59,100]; see Figure 1 above for a schematic depiction). Leaves must import sufficient water to compensate for water lost during photosynthetic CO_2_ uptake and keep the stomates open [11,12]. On the other hand, the expedient removal of sugar produced in photosynthesis is important to avoid carbohydrate back-up in leaves and photosynthetic downregulation by sugar signals [8,101,102].

#### 4.5.2. Upregulation of Sugar-Export Capacity under Cool Growth Temperature

Challenges to photosynthetic activity under cool temperature include reduced enzyme activity and resulting decreases in the carbon-fixation rate and in transporter-dependent loading of sugar into sugar-export conduits as well as increases in phloem-sap viscosity. The cool-temperature-induced limitations to foliar sugar export (and feedback inhibition of photosynthesis) can be overcome by upregulation of a suite of biochemical and infrastructural features [75,103] that support sugar-export capacity and prevent the repression of photosynthetic genes. In other words, limiting photosynthate-export capacity from a source leaf can be thought of as a bottleneck and a type of sink limitation close to home.

**Figure 7:**
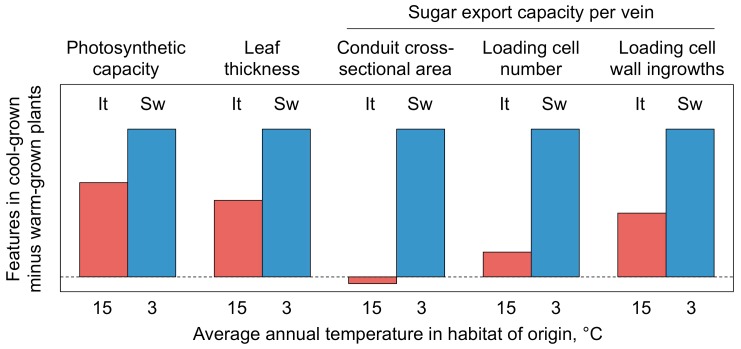
Differences in leaf and minor-vein features of Italian (It) and Swedish (Sw) ecotypes of *A. thaliana* grown at cool (14 °C) versus warm (36 °C) leaf temperatures under a moderate light intensity of 400 µmol photons m^−2^·s^−1^, in relation to average annual temperatures of the locations from which the ecotypes originated. Based on data from Adams et al. [75].

**Figure 8:**
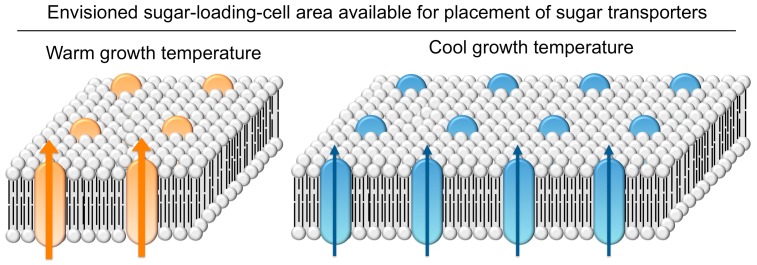
Schematic depiction of an increased membrane surface area of sugar-loading phloem cells, with a greater number of sugar transporters and ATPases that can counteract the reduced activity of such transporters (indicated by arrow width) in winter annual apoplastic loaders at cool versus warm growth temperatures. Based on data from Adams et al. [75,103].

*Arabidopsis thaliana* leaves load sugars into sugar-exporting phloem conduits via membrane transporters (see [104]). An increased loading-cell surface area can accommodate more membrane transporters and thereby counteract the inhibitory effect of decreasing temperature on transporter activity. When grown under cool temperature, *A. thaliana* upregulates the number of sugar-loading cells as well as phloem-cell-wall ingrowths that magnify cell membrane area in concert with photosynthetic capacity (Figure 7). The greater upregulation of loading-cell number and cell-wall ingrowths in the Swedish versus the Italian ecotype can serve as a proxy for a greater upregulation of loading-cell surface area (Figure 8; [75]).

In addition, to compensate for increased viscosity of the sugar-laden sap exported from leaves at low temperature (see Discussion in [105]), the combined volume of sugar-export conduits can be increased, which helps avoid foliar sugar accumulation and photosynthetic downregulation [101,102]. Figure 7 shows greater upregulation of sugar-conduit cross-sectional area, as a proxy for sugar-export-conduit volume, in the Swedish versus the Italian ecotype under cool versus hot growth temperature ([75]; see also [76]). These adjustments can support the maintenance of photosynthesis rate under prevailing cool temperature and result in record rates of photosynthesis under warmer temperatures at midday.

#### 4.5.3. Upregulation of Foliar Water-Import Capacity under Hot Growth Temperature

The maintenance of high photosynthetic productivity under hot temperature requires increased water delivery to the leaf to compensate for enhanced evaporative water loss and support continued stomatal opening and carbon gain. This link between water delivery to leaves and stomatal opening is described in models on the hydraulic efficiency-photosynthesis connection [11,12,106]. In *A. thaliana* grown under hot temperature, water delivery to leaves is increased via increased foliar vein density and an increased ratio of water-import to sugar-export capacity, accompanied by an increased ratio of transpiration to photosynthesis [71,75,76].

The transcription factor DREB2C, implicated in thermotolerance in *A. thaliana* [26,107], is most heavily expressed in the vasculature in response to heat stress [108], and may therefore play a role in the upregulation of foliar water-import capacity. A particularly pronounced increase in vein density and in the number of water conduits per vein under hot growth temperature was seen in an *A. thaliana* ecotype from a location with relatively low levels of annual precipitation [75,76]. This adaptation strategy is unique to annual species. In contrast, woody perennials typically restrict water delivery to the leaves under drought/heat through development of narrower conduits that are less susceptible to cavitation. For example, in a study on a range of woody perennials, Blackman et al. [109] found leaves with narrow, thick-walled water conduits to be advantageous under drought stress. Moreover, Pfautsch et al. [110] found a strong inverse relationship between water-conduit diameter and aridity across multiple *Eucalyptus* tree species in Australia.

### 4.6. Leaf Acclimation to Light Environment Defines Sun-Loving and Shade-Loving Genotypes

#### 4.6.1. Adjustment of Leaf Size and Thickness

Plant acclimation to high-light environments typically involves thicker leaves [111]. In such environments, enough light is available to penetrate into the lower expanses of thick leaves that display chloroplasts and chlorophyll in a vertical column and require high light intensities for light saturation of photosynthesis of the self-shaded chloroplasts in the lower portion of the leaf. A survey among tree species found that sun-loving species exhibited greater upregulation of photosynthesis, leaf thickness, and hydraulic conductivity under high compared to low growth light intensity [112].

Conversely, larger leaf areas are often seen in plants acclimated to shaded environments [111,113]. Larger leaves allow for display of chloroplasts and chlorophyll horizontally, which minimizes competition for light by self-shading. Interspecies comparison revealed similar differences between shade- and sun-loving species, with greater leaf areas per plant and greater absolute growth rates in low light in highly shade-tolerant species [114].

The differential trends in acclimation to light environment seen in the ecotype pair from Sweden and Italy identify the Swedish ecotype as sun-loving and shade-intolerant and the Italian ecotype as shade-tolerant. Just as was seen under hot versus cool growth temperatures [76], the Swedish ecotype did not turn off the cool-temperature/high-light phenotype as effectively as the Italian ecotype under low growth light intensity either (Figure 6; [86]). Low-light-grown plants of the Swedish ecotype still had somewhat thicker leaves and much smaller rosettes than the Italian ecotype.

#### 4.6.2. Adjustment of Leaf Vascular Anatomy

The volume of both water and sugar conduits was greater in leaves grown under high versus low light [71,86]. This is expected since higher photosynthesis rates in a high-light environment create a greater demand for both sugar export and water import. Once again, the degree of this acclimation was more pronounced in the Swedish versus the Italian ecotype. Specifically, high-light-grown leaves of the Swedish ecotype exhibited a greater photosynthetic capacity and greater leaf mass per area, a greater number of chloroplast-rich palisade cell layers, as well as greater numbers of sugar-loading phloem cells, larger sugar-export conduits, and larger total volumes of water conduits [86]. Conversely, the Italian ecotype exhibited superior acclimation to low growth light intensity, with larger rosette areas and greater aboveground biomass accumulation in low-light-grown plants [86,87].

### 4.7. What Is Known about the Regulators of Vascular Organization

Little is known about an involvement of CBF/DREB in leaf vascular organization, except for *DREB2C* expression in the vasculature under heat stress (see above). Future studies should give attention to a possible DREB involvement in a putative greater xylem-to-phloem capacity in drought-tolerant crop varieties that exhibit larger root volumes, greater access to water in the soil, and higher stomatal conductance and photosynthesis rates than drought-sensitive varieties [115].

As discussed above, the CBF/DREB regulon includes many genes involved in various aspects of temperature adaptation and interacts strongly with phytohormones and redox messengers. Mutant studies have revealed involvement of both phytohormones and antioxidants in the modulation of vascular development. For example, auxin plays a major role in leaf venation patterning [116,117]. Foliar vein density, hydraulic conductance, and CO_2_ and water exchange was reduced in auxin-deficient mutants compared to wild-type [118]. Vascular acclimation also responds to tocopherol level [69]; tocopherol deficiency was associated with an increased proportion of water to sugar conduits at high temperature, as well as higher vein density under some growth conditions [71,72].

## 5. From Mechanistic Insight to Crop Improvement

We hope that the insight summarized here will contribute to future efforts in crop improvement. Combining eco-physiological work with “-omics” tools and targeted gene editing should also be able to address the questions posed here. Such integrative approaches can be added to the many ongoing efforts to improve plant performance in stressful environments using other key regulators, such as stress-responsive NAC (SNAC) [119], homeodomain (HD)-START [120], nuclear factor-Y (NF-Y) [121], and HARDY [122] transcription factors (for a review of these transcription factors as well as CBFs/DREBs in the context of drought stress tolerance, see [123]).

## 6. Conclusions

1. To provide guidance for future germplasm screening, genomics-directed breeding, and gene editing, studies into adaptive traits should give attention to the whole-plant source–sink relationship and a range of relevant environmental conditions.
2. Superior plant productivity depends on distinct plant features in different environments, and could be custom-designed for specific local contexts using environmentally induced gene regulators, such as CBF/DREB transcription factors, that coordinate multiple aspects of plant function.
3. *Arabidopsis thaliana* remains an ideal model organism. In terms of eco-physiology, it occupies a range of native habitats, with different ecotypes manifesting distinct functionality under controlled growth conditions. On the molecular side, over a thousand sequenced *A. thaliana* genomes are available for transformation of selected ecotypes via precision gene editing.

## Figures and Tables

**Table 1:** Anatomical features of water-transporting xylem conduits in the leaves of wild-type (Col-0) *A. thaliana* and a tocopherol-deficient mutant (*vte1*) grown at hot temperatures (36 °C).

	Water Conduit Number per Vein *	Individual Water Conduit Cross-Sectional Area, µm^2^ **
Col-0 wild-type	8.0 ± 0.5	23.8 ± 1.7
Tocopherol-deficient *vte1* mutant	10.5 ± 0.4	20.5 ± 0.6

Mean values ± standard errors (*n* = 4 to 5); significant differences (*t*-test) indicated by asterisks—* = *p* < 0.05, ** = *p* < 0.01. Data from Stewart et al. [71].

## Abbreviations

CBF	C-repeat binding factor
CT	cool growth temperature
ΔpH	trans-thylakoid pH gradient
DREB	dehydration-responsive element-binding factor
HL	high growth light intensity
HT	hot growth temperature
LL	low growth light intensity
NPQ	non-photochemical quenching of chlorophyll fluorescence
ROS	reactive oxygen species
